# Usefulness of unwinding the colonoscope shaft loop via the universal cord for colorectal endoscopic resections

**DOI:** 10.1002/ccr3.9461

**Published:** 2024-09-20

**Authors:** Taiji Yoshimoto, Hiroshi Takihara, Ryuichi Yamamoto

**Affiliations:** ^1^ Department of Gastroenterology Musashino Tokushukai Hospital Tokyo Japan; ^2^ Department of Gastroenterology Uji Tokushukai Hospital, Uji Kyoto Japan; ^3^ Department of Gastroenterology Tokyo‐west Tokushukai Hospital Tokyo Japan

**Keywords:** endoscopic colorectal resections, endoscopic mucosal resection, endoscopic submucosal dissection, polypectomy

## Abstract

The colonoscope shaft loop can be unwound by establishing a loop in the universal cord of the colonoscope to maintain the same endoscopic view during colorectal endoscopic resections.

## 1 INTRODUCTION

Endoscopists must rotate the endoscope to bring the lesion closer to the forceps hole (typically in a 5–6 o'clock direction) while performing endoscopic colorectal resections.^1^, ^2^ During this process, a loop is sometimes created in the colonoscope shaft and the endoscopist's hands frequently need to maintain the loop to keep the same endoscopic view, making free hand movement difficult.

**FIGURE 1:**
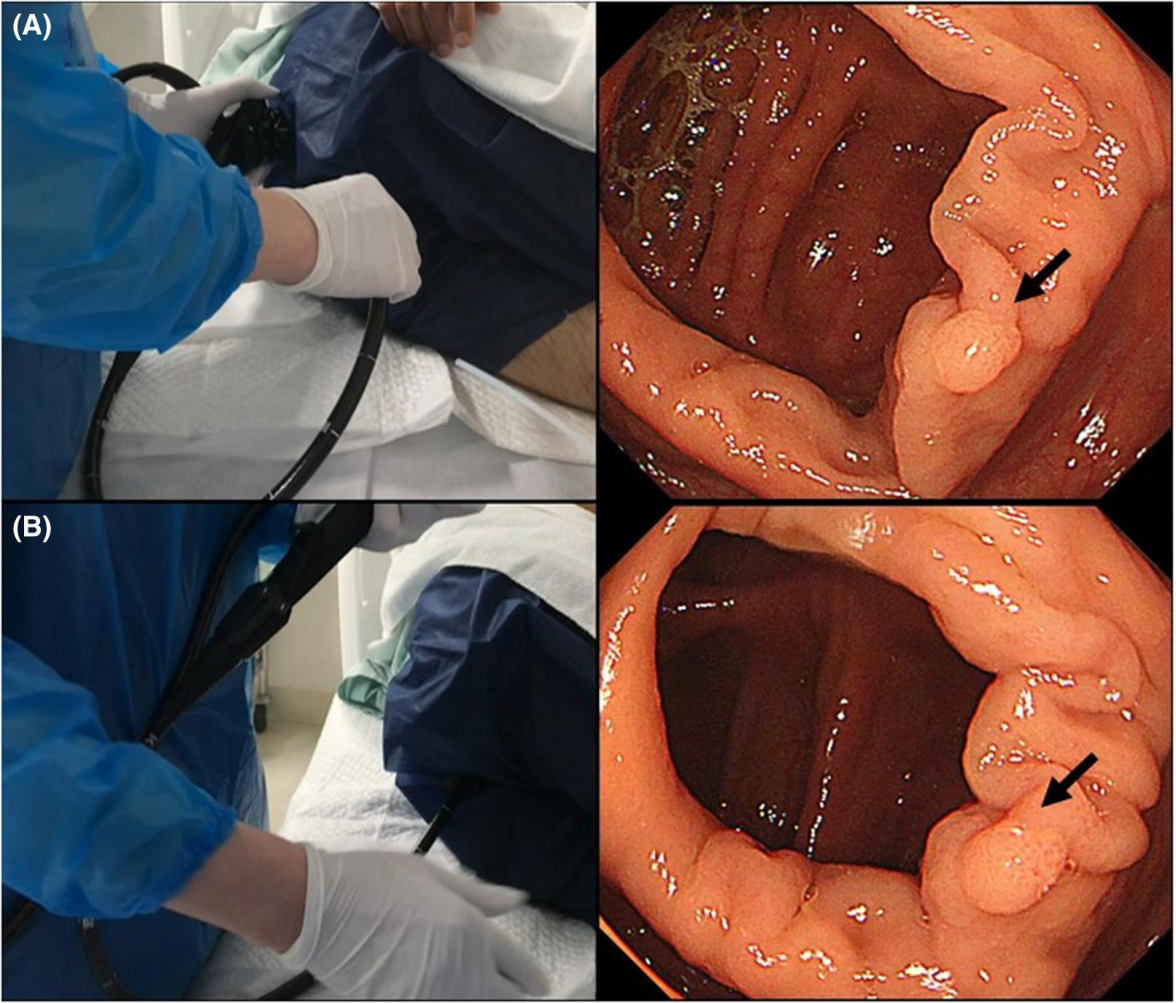
Colonic polypectomy case depicting the unwinding usage of the colonoscope shaft loop via the universal cord. Polyp of macroscopic type 0–Is, 5 mm in size, and located in the proximal ascending colon; arrow indicates the polyp. (A) Endoscope must be rotated counterclockwise to bring the polyp to the 5 o'clock direction, restricting the endoscopist's hand movement. (B) After unwinding the colonoscope shaft loop via the universal cord, the polyp remains in the 5 o'clock direction; however, the endoscope returns to a neutral position, enabling free hand movement.

Unwinding the colonoscope shaft loop via the universal cord can be used for colorectal endoscopic treatment, such as colorectal endoscopic resections, because it frees the endoscopist's hands while maintaining the same endoscopic view during colorectal endoscopic resections (Figure 1, Video 1).

**VIDEO 1:** Colonic polypectomy case using the unwinding of the colonoscope shaft loop via the universal cord.

## AUTHOR CONTRIBUTIONS

**Taiji Yoshimoto:** Conceptualization; resources; supervision; writing – original draft; writing – review and editing. **Hiroshi Takihara:** Conceptualization; supervision; writing – review and editing. **Ryuichi Yamamoto:** Conceptualization; resources.

## FUNDING INFORMATION

No funding was received.

## CONFLICT OF INTEREST STATEMENT

The authors have no conflicts of interest to declare.

## CONSENT

Written informed consent was obtained from the patient to publish this report in accordance with the journal's patient consent policy.

## Data Availability

The data that support the findings of this study are available from the corresponding author upon reasonable request.

## References

[1] Yoshimoto T , Takihara H , Yoshihara T , et al. Usefulness of “Nelaton attachment” for endoscopic submucosal dissection of colorectal neoplasms. Endosc Int Open. 2019;7(9):E1187‐E1191. 31475238 10.1055/a-0961-7542 PMC6715429

[2] Hsu CW , Wu CC , Lee MH , Wang JH , Chen YH , Chang MC . Endoscope rotating technique is useful for difficult colorectal endoscopic submucosal dissection. Surg Endosc. 2020;34(2):1006‐1011. 31482351 10.1007/s00464-019-07105-1

